# Imaging of complications following Fontan circulation in children — diagnosis and surveillance

**DOI:** 10.1007/s00247-020-04682-5

**Published:** 2020-05-28

**Authors:** Charlotte de Lange

**Affiliations:** 1 Department of Radiology and Clinical Physiology, Queen Silvia Children’s Hospital, Rondv.10, S-41516 Gothenburg, Sweden; 2 Department of Radiology and Nuclear Medicine, Oslo University Hospital, Oslo, Norway

**Keywords:** Child, Computed tomography, Elastography, Fontan circulation, Heart disease, Magnetic resonance imaging, Surveillance, Ultrasonography

## Abstract

The Fontan operation is performed for various cardiac lesions with single-ventricle physiology. The survival rate of Fontan patients is increasing for adolescents and young adults, with an expected 30-year survival of >80%. Medical health care providers including specialists in organ systems and pediatric radiologists need to improve their knowledge about the Fontan circulation and the various organ complications to monitor care. In this review the author explains the basic anatomical and functional features of Fontan palliation and gives an overview of the multiple long-term organ complications that might present in the pediatric population. These include decreased physical capacity, ventricular dysfunction, atrioventricular valve regurgitation and arrhythmia, as well as protein-losing enteropathy, plastic bronchitis, growth/bone composition disturbances, renal dysfunction, and the recently recognized and important liver fibrosis (Fontan-associated liver disease). Neuropsychological and behavioral deficits occur frequently. This review focuses on the key role of radiology in making the diagnosis of these complications, monitoring therapy and predicting outcomes in the pediatric age group. The author discusses how and when radiology is important in Fontan patients, as well as how new techniques enabling quantitative measures in imaging with US, MRI and CT are adapted for pediatric use, and how they contribute to urgently needed surveillance strategies.

**Electronic supplementary material:**

The online version of this article (10.1007/s00247-020-04682-5) contains supplementary material, which is available to authorized users.

## Introduction

Four decades after the first operation performed by Francis Fontan, much is still unknown of the pathophysiological mechanisms and complications of Fontan palliation as a lifesaving treatment for single-ventricle congenital heart disease. The ventricular function alone is not predictive for a Fontan patient’s quality of life and the multiple types of problems can interact to give the unique features of complications in this equally exclusive and growing population. These children require lifelong follow-up with medical care, implicating an important financial and societal impact.

**Fig. 1:**
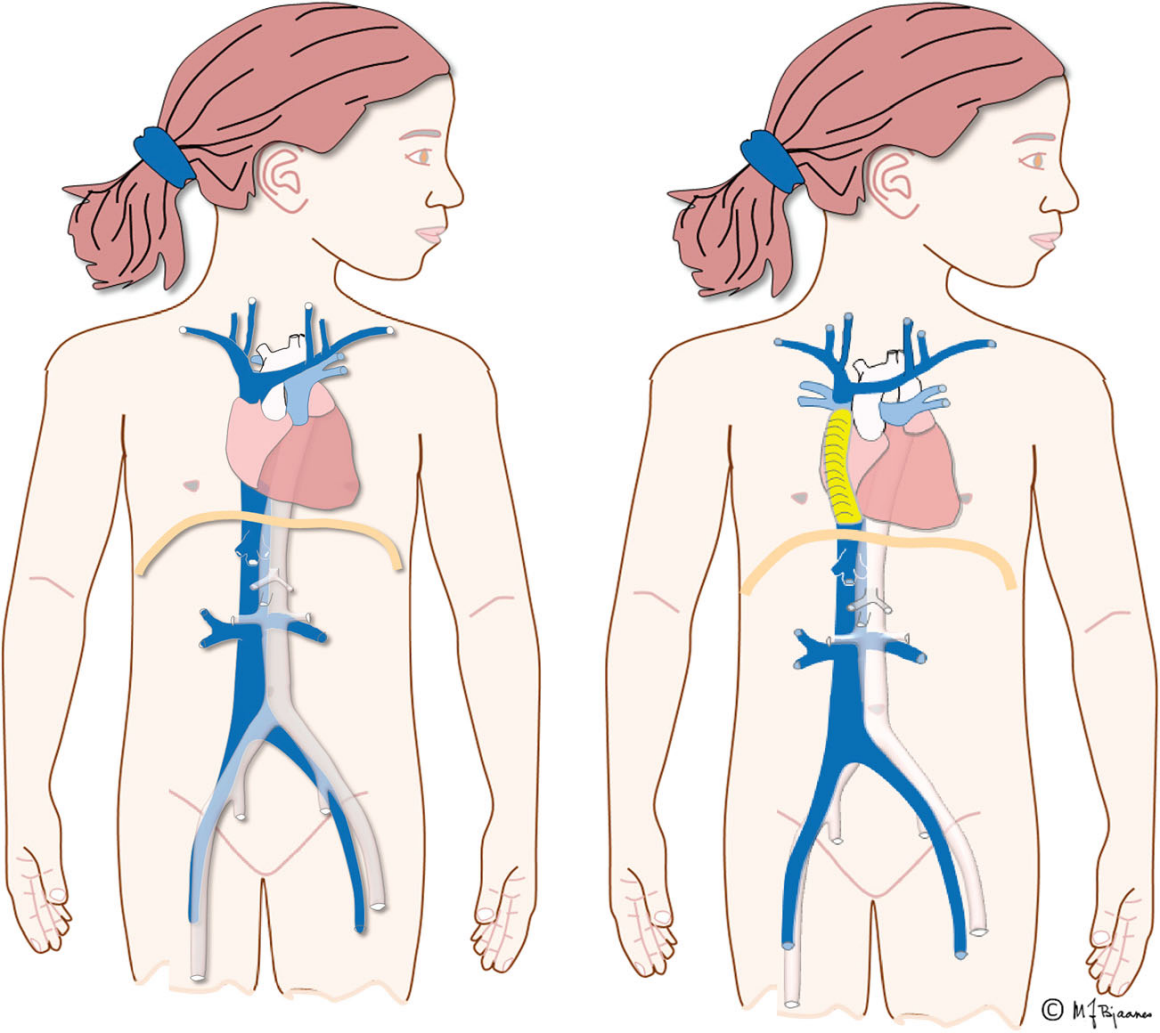
Illustrations of normal a heart circulation and a Fontan circulation in two girls. Left, a normal heart with pulmonary and arterial circulation in parallel. Right, a girl with a total cavo-pulmonary connection with an extracardiac conduit (*yellow*), the Fontan circulation, where the pulmonary and arterial circulations are serial and bypass the right ventricle. The inferior vena cava is enlarged because of increased central venous pressure. Illustrations created by Michael Bjaanes in collaboration with the Department of Pediatric Cardiology at Oslo University Hospital

**Fig. 2:**
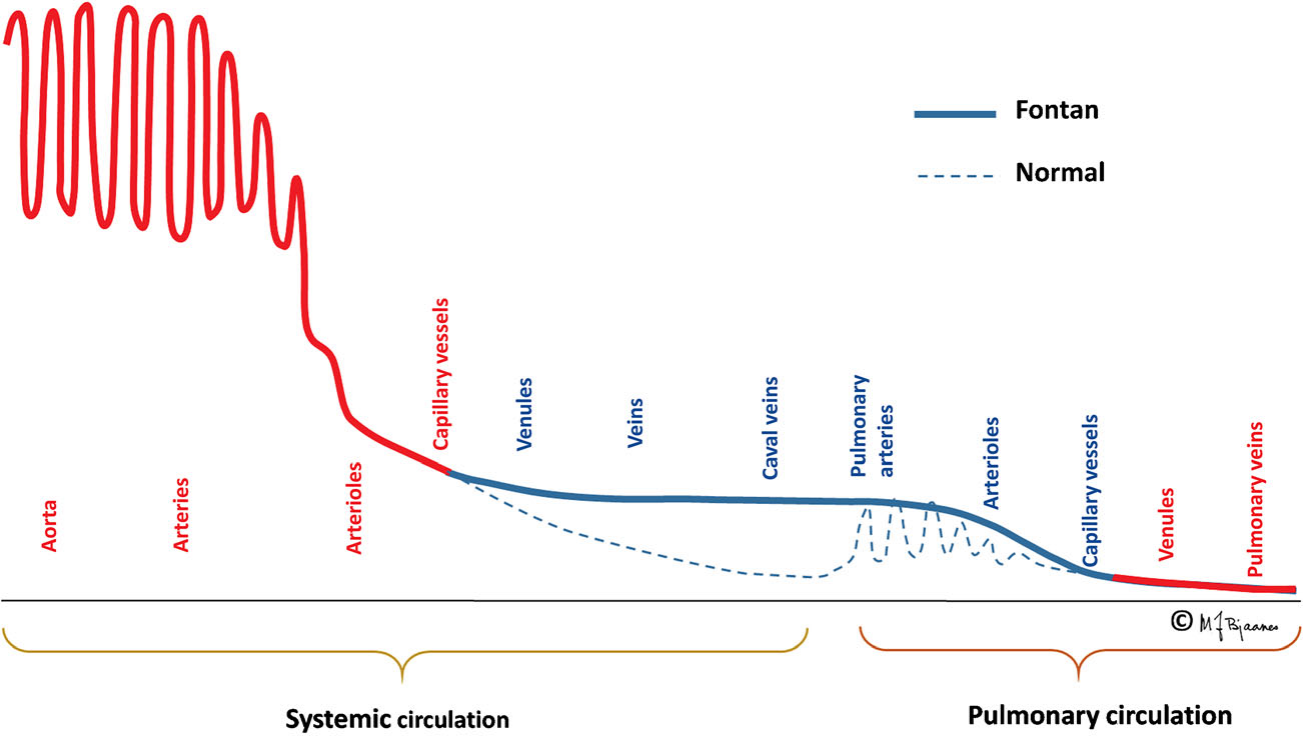
The graphic depicts blood pressure differences in a normal and Fontan circulation. Illustrations created by Michael Bjaanes in collaboration with the Department of Pediatric Cardiology at Oslo University Hospital. Reprint of modified version with permission from [2]. Red: oxygenated blood, blue: deoxygenated blood

The Fontan operation creates an artificial circulation with two serial capillary beds by connecting the superior and inferior caval veins to the pulmonary arteries (Fig. 1) [1]. In the final Fontan circulation, transpulmonary blood flow is driven by a moderately elevated central venous pressure high enough to overcome the pulmonary vascular resistance (Fig. 2) [2]. The most important elements of Fontan circulation include:

- reduced cardiac output,
- increased central venous pressure and
- non-pulsatile hepatic congestion and fibrosis/cirrhosis.

**Fig. 3:**
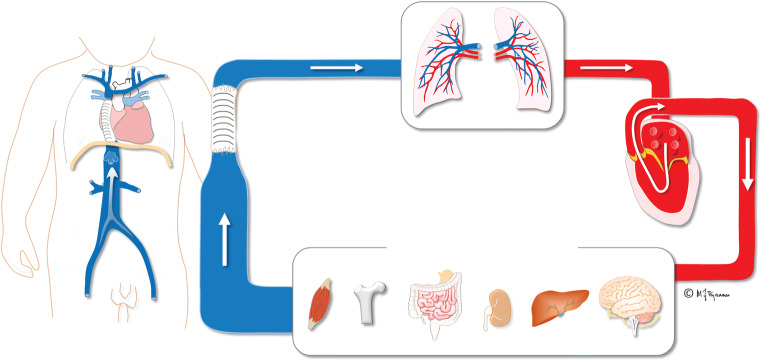
Illustration of Fontan circulation. Serial circulation where the single functional ventricle pumps arterial blood to the systemic circulation and the organs. The systemic venous blood from the inferior and superior caval veins drains with a non-pulsatile low pressure into the pulmonary arteries and lungs. Illustration created by Michael Bjaanes in collaboration with the Department of Pediatric Cardiology at Oslo University Hospital. Reprint of modified version with permission from [2]. Red: oxygenated blood, blue: deoxygenated blood

These features have an impact on both visceral organs and lymphatic drainage, causing important complications (Fig. 3) [2–5]. One of the most important effects that is lately gaining more awareness is Fontan-associated liver disease with development of fibrosis/cirrhosis and risk for malignant transformation. Liver disease is of major concern in young adult Fontan patients, whereas the prevalence and impact in the pediatric population is less well explored [6–8].

The cardiac complications with decreased cardiac output go along with decreased physical capacity, ventricular dysfunction, atrioventricular valve regurgitation and arrhythmia. The veno-lymphatic congestion with protein-losing enteropathy and plastic bronchitis are a considerable problem for some patients, as well as renal dysfunction, growth disturbances and bone composition abnormalities that can progress over time. Neurodevelopmental disorders and psychological problems are highly prevalent.

Imaging is gaining an important role in the follow-up of this patient group and, most important, in the evaluation of cardiac, lymphatic and liver function. Cardiac and thoracic CT and cardiac MRI are used to supplement or replace echocardiography and cardiac catheterization for assessing the cardiac status. In addition, with the increasing awareness of other end-organ injuries, new techniques in US, CT and MRI such as elastography, relaxometry with quantitative measures, and lymphatic imaging with image-guided interventions have become increasingly important.

The question is whether and how these children should be followed appropriately to identify treatable complications. One study suggests that with a growing adult population with congenital heart disease, adult congenital cardiologists should assure the transition from pediatric to adult care actively and start this process during early adolescence, continuing until successful engagement [9].

The involvement of multiple organs in Fontan circulation is challenging to manage and requires a multidisciplinary approach by those with medical knowledge of the pathophysiological origin to achieve optimal care for this distinct condition. This includes clinicians from many pediatric and adult specialties, including hepatology, pneumology, endocrinology, cardiology and radiology.

## Incidence, prognosis and outcome

Up to 70,000 people with Fontan-type palliation might be alive worldwide, and the population is expected to double the next 20 years [5, 10, 11]. The long-term survival is increasing because of surgical refinements and improved pre-, peri- and postoperative care, but this varies among countries. Today, 20-year survival estimates are 61–85% [2, 11–14]. Known factors negatively influencing outcomes are multiple, such as having a single right ventricle, male gender, common atrioventricular valve, older age at Fontan operation, postoperative complications with increased pulmonary pressures, and prolonged pleural effusions after Fontan completion [3]. Likewise, protein-losing enteropathy, arrhythmia and thromboembolic events are associated with shorter survival.

The Fontan circulation is a palliation, and cardiac transplantation — the only curable treatment for a failing Fontan circulation — has important implications for survival. The paediatric heart network reported 15-year survival results ranging from 0% to 21% across centers among those who received a cardiac transplant after Fontan surgery completion [15].

## Pathophysiology

**Fig. 4:**
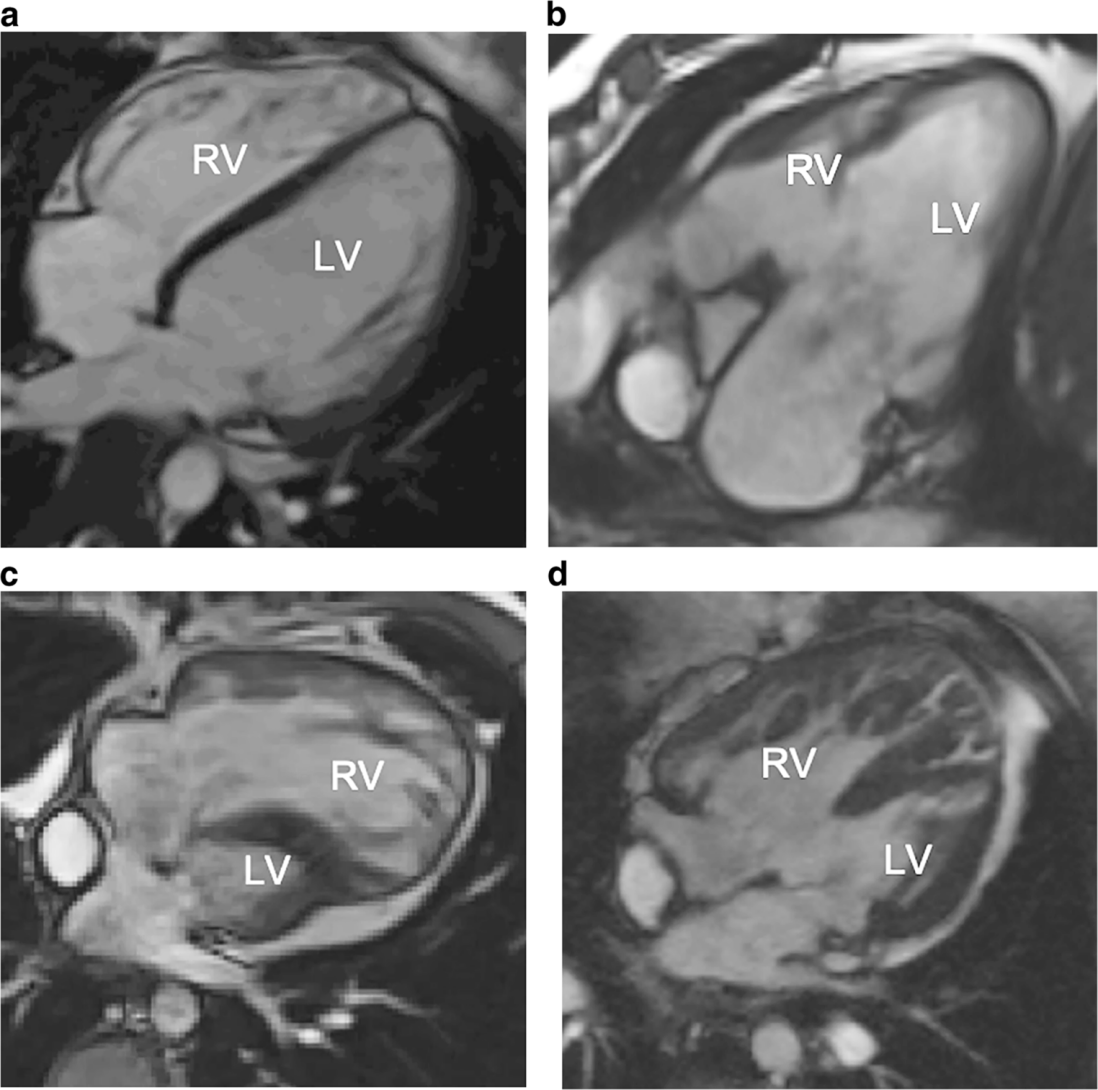
Cardiac anomalies with indication for Fontan procedure. **a–d** Cardiac MRI, 2-D steady-state free precession sequence, four-chamber view in (**a**) a normal 17-year-old boy, (**b**) a 16-year-old girl with tricuspid atresia with a small right ventricle, (**c**) a 15-year-old boy with hypoplastic left heart syndrome with a small left ventricle and (**d**) a 14-year-old girl with double-outlet right ventricle with a large ventricular septal defect with a slightly smaller right ventricle. *LV* left ventricle, *RV* right ventricle

The most common type of heart defect treated with a Fontan operation is hypoplastic left heart syndrome. However, in several other defects with a single functional ventricle such as tricuspid atresia, double-outlet right ventricle, some types of pulmonary atresia, and atrioventricular septum defects, the only treatment option might be the Fontan palliation. In many cases, either the left or the right ventricle is dominant, serving as the systemic ventricle (Fig. 4).

The goal with a Fontan palliation is to create a serial circulation where the systemic venous return via the superior and inferior caval veins reach the pulmonary vascular bed without the pumping pressure of the right ventricle. Instead, systemic venous blood return is driven by increased central venous pressure and without pulsations (Fig. 1).

This passive blood flow does, however, limit the ventricular volume load and thereby limit the possibility of increasing cardiac output during physical activity (Fig. 2). The non-pulsatile flow additionally causes injury of pulmonary vessel walls with elevated pulmonary vascular resistance. Combined with limited capacity of ventricular filling, this leads to a circulatory failure, but not necessarily combined with a failing ventricular pump function [4]. As a compensation, venovenous, aortopulmonary and even pulmonary arteriovenous collaterals develop at the cost of desaturation and cardiac volume overload.

## Surgical treatment

**Fig. 5:**
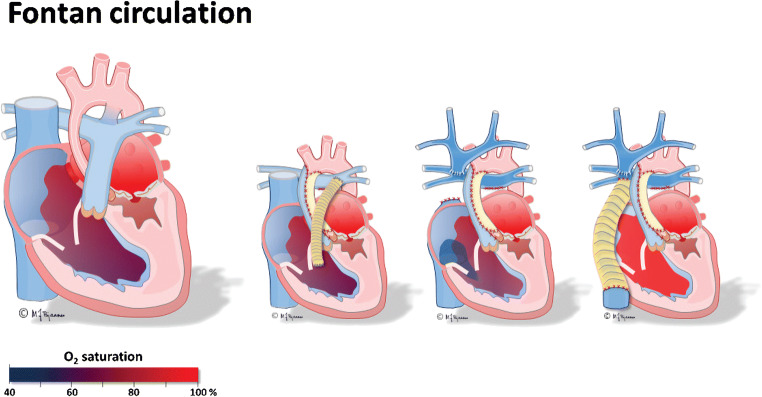
Fontan circulation from first to final stages of total cavo-pulmonary connection, illustrated in the hypoplastic left heart syndrome. Illustration of untreated hypoplastic left heart syndrome is to the left, followed by the three surgical operations: Step 1, a shunt (homograft-patch, *orange*) is created from the single right ventricle to the pulmonary artery and the atrial septal defect is enlarged. Step 2, a bidirectional cavo-pulmonary anastomosis is created while the shunt is removed. In the third and final step, the extracardiac conduit in Gore-Tex (*yellow*) is placed as a total cavo-pulmonary anastomosis, creating the Fontan circulation. An animation of the blood flow in the different surgical stages during the myocardial contraction can be seen in the supplementary material. Illustrations created by Michael Bjaanes in collaboration with the Department of Pediatric Cardiology at Oslo University Hospital. Reprint of modified version with permission from [2]

The surgical repair is generally performed in planned stages (Fig. 5). The first operation is carried out when necessary shortly after birth to assure sufficient arterial flow and oxygenation to the lungs and body. This operation might differ depending on the type of defect, but in hypoplastic left heart syndrome, a Blalock–Taussig shunt or a central aorto-pulmonary shunt is placed and one common atrium created by a large interatrial defect to secure free return of pulmonary and systemic venous flow (Fig. 5). The following stage of transitional superior cavo-pulmonary connection (either a bidirectional Glenn or cavo-pulmonary connection) is performed at about 6 to 8 months of age. The operation for the final stage, a total cavo-pulmonary connection, is accomplished in most centers when the child weighs about 15 kg, at 2–4 years of age (Fig. 5) [16].

Historically, three main surgical techniques for the final Fontan stage have been used and continuously refined over the years. The initial type was introduced by Francis Fontan in 1971 and was further refined by Guillermo Kreutzer in 1973 using the “atriopulmonary connection,” where the right atrial appendage connects to the left pulmonary artery [1, 17]. In the second, “the lateral tunnel approach,” a tunnel inside the atrium creates a connection to the pulmonary artery to achieve a total cavo-pulmonary connection.

Most children today undergo a third technique, using an extracardiac conduit in Gore-Tex placed lateral to the right of the common atrium between the inferior caval vein and the left pulmonary artery (Fig. 5 and supplementary material) [18]. Some centers perform a fenestration between the conduit and the common atrium to temporarily reduce the central venous pressure, i.e. a controlled right-to-left shunt, at the cost of increased cyanosis. This seems to decrease the amount of immediate postoperative pleural effusion, and the fenestration might eventually close naturally or be closed later [19].

## Complications of Fontan circulation and the role of imaging

The Fontan circulation is a life-saving procedure providing many children an acceptable quality of life for several years [20]. However, long-term complications occur, and with increasing severity from adolescence (Table 1; Fig. 3) [5, 6, 21–25]. In single-ventricle physiology, the hemodynamic situation is abnormal already in fetal life, with deprived oxygenation that is detrimental to body organs and further develops after Fontan completion (Table 1; Fig. 3) [24]. Ultimately, this can lead to a “failing Fontan circulation,” a term traditionally used when several and severe complications are present and can lead to death [26]. It does not necessarily include a circulatory failure caused by ventricular dysfunction, but it can be the result of pulmonary vascular dysfunction, thromboembolism, organ failure or arrhythmia. Most imaging techniques using echocardiography, radiography, US, CT, MRI and conventional angiography are applicable for investigating the complications in the heart and in secondary findings in the thorax and end organs.

**Table 1:** Long-term complications of Fontan circulation and the appropriate imaging techniques to investigate and monitor them

Organ system	Complication	Radiologic investigation, monitoring
General	Reduced physical capacity (present in 100% of patients)	
Cardiac	Ventricular dysfunction	Echocardiography/cardiac MRI/CT
	Atrioventricular valve regurgitation Arrhythmia	
	Heart failure	
	Cyanosis	
Lungs	Increased pulmonary vascular resistance	CT/catheterization Angiography/cardiac MRI
	Aortopulmonary/venovenous collaterals	
Liver	Fibrosis/cirrhosis/hepatocellular carcinoma	US, CT, MRI
Kidney	Renal dysfunction	Doppler US
Veno-lymphatic system	Lymphangiectasia	MR, lymphangiography
	Plastic bronchitis	MR/CT/lymphangiography
	Protein-losing enteropathy	Catheterization intervention
Bone	Growth disturbances	Radiographic bone age estimation
	Bone mineral deficiency	Bone mineral density measurement
Brain	Neurodevelopmental disorders	MR brain
	Cognitive disorders	
	Behavioral deficits	

*CT* computed tomography, *MRI* magnetic resonance imaging, *US* ultrasonography

## Cardiovascular complications

### Heart failure and ventricular dysfunction

**Fig. 6:**
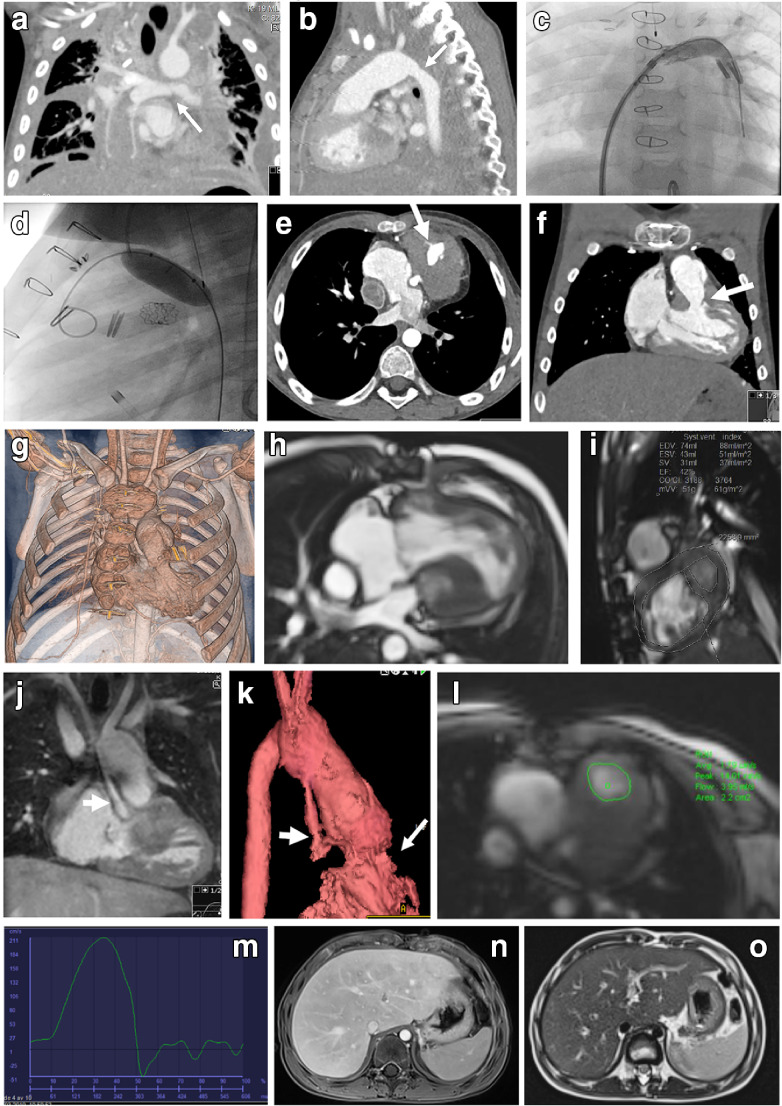
Cardiovascular complications of Fontan palliation in a boy with hypoplastic left heart syndrome. **a–d** CT angiography at 4 years old. Coronal (**a**) and sagittal (**b**) early postoperative images after Fontan completion show residual pleural effusions, stenosis of the left pulmonary artery (*arrow* in **a**) and narrowing of the aortic arch anastomosis (*arrow* in **b**). Angiography with balloonplasty of the left pulmonary artery shows a stent (**c**) and the aortic arch anastomosis (**d**). **e–g** Five years later at 9 years of age, the boy presented with echocardiographic findings of a subaortic stenosis confirmed on CT angiography in axial (**e**), coronal (**f**) and volume-rendered reconstruction (**g**) images, revealing the subaortic stenosis (*arrows*) caused by muscular hypertrophy. **h, i** Additional functional information from cardiac MRI. Cine 2-D steady-state free precession four-chamber (**h**) and short-axis (**i**) views show volumetric measurements. **j–m** Contrast-enhanced MR angiography in coronal (**j**) and volume-rendered (**k**) images. Note the thin native aorta (*thick arrows*) and the subaortic stenosis (*thin arrow* in **k**). Through-plane velocity-encoded phase-contrast measurement at the subaortic level (**l**) reveals a moderate increased velocity and a small regurgitation (**m**). **n, o** Consecutive MR exam of the liver. Axial contrast-enhanced T1-W gradient echo sequence (**n**) and T2-weighted sequence (**o**) reveal a slightly enlarged liver with peripheral enhancement pattern and perisinusoidal sparing, consistent with liver congestion

There are two types of heart failure in Fontan patients. They present as the classic ventricular pump failure and the Fontan circulatory failure. The latter relates to the chronic increased central venous pressure and low cardiac output but with a remaining acceptable ventricular function. The systolic and diastolic ventricular function are relatively preserved the first decades after Fontan completion but decline over time [15]. Systolic dysfunction is present in 40–60% of patients considered for heart transplantation [27]. The function might be improved if treatment is possible for coexisting complications such as atrioventricular valve regurgitation, arrhythmia, venovenous and arteriovenous collaterals, and vascular obstructions like stenosis (Fig. 6). Reliable quantification of the function is difficult in single-ventricle physiology because of the complex diverse anatomy and lack of normal reference values and functional indexes for a non-left ventricular systemic ventricle [28].

### Atrioventricular valve regurgitation

Historically, significant atrioventricular valve regurgitation was a contraindication for the final Fontan operation, but today medication and options to replace or repair the valve are possible. However, it is still an important complication with often insidious development that can lead to volume overload, ventricular dilation and dysfunction. This can compromise the Fontan circulation, especially in a tricuspid valve serving as a systemic valve [29]. Preoperative evaluation and quantification of ventricular function and valve regurgitation are important to decide the optimal surgical approach and time point [30, 31]*.*

### Arrhythmias

Both the anatomy of the single-ventricle congenital heart defect and the type of surgery performed pose a risk for arrhythmia, which occurs in about 41% of Fontan patients [32]. Arrhythmias are associated with increased mortality, morbidity and atrial/ventricular arrhythmia focus (90/10%) [32]. Sinus node dysfunction is predominant in children with extracardiac conduit. Treatment options include antiarrhythmic medication and catheter-based ablation, different modes of pacing or, in specific cases, an implantable cardioverter defibrillator.

### Thromboembolic events

**Fig. 7:**
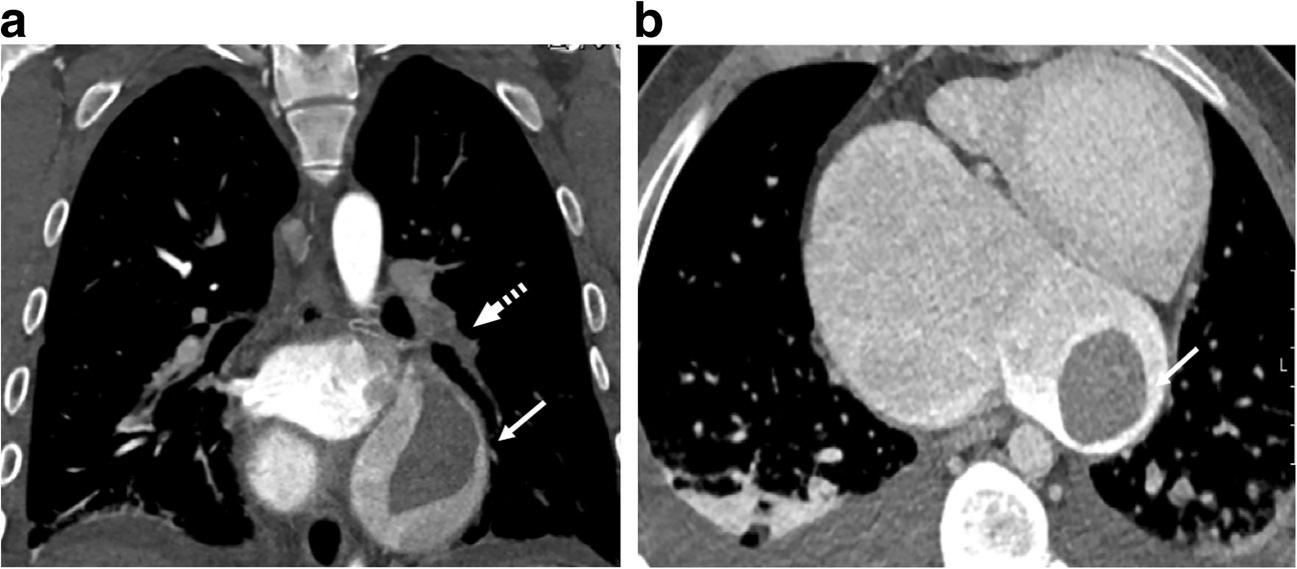
Thromboembolic event in an 18-year-old male Fontan patient with tricuspid and pulmonary atresia. **a, b** CT angiography. Coronal (**a**) and axial (**b**) images reveal a thrombus on the left side of the atrium (*solid arrows*), pulmonary embolus in the left lower branch of the pulmonary artery (*dashed arrow*) and bilateral pleural effusion

Fontan patients have increased risk of developing thrombosis and bleeding. The chronic low oxygen saturation in Fontan patients, about 90–95% at rest, leads to increased hemoglobin and blood viscosity. Together with venous stasis, a low cardiac output and indwelling central lines lead to a hypercoagulable state. On the other hand, intrinsic alterations in thrombophilia factors increase the risk for bleeding. There is no consensus on the use of thromboprophylaxis, which leaves up to one-third of patients at risk of a thromboembolic event. Pulmonary embolism is one of the major mortalities in Fontan patients (Fig. 7) [33].

### Imaging in cardiovascular complications

Two-dimensional (2-D) echocardiography is the easiest and first choice of investigation for functional and anatomical examination, with new techniques enabling volumetric measurements, 3-D and 4-D imaging, myocardial strain analysis, and feature tracking [34]. However, interpretation is partly subjective and often hampered, especially for the right ventricle, by limited cardiac views related to patient habitus and postoperative conditions.

**Fig. 8:**
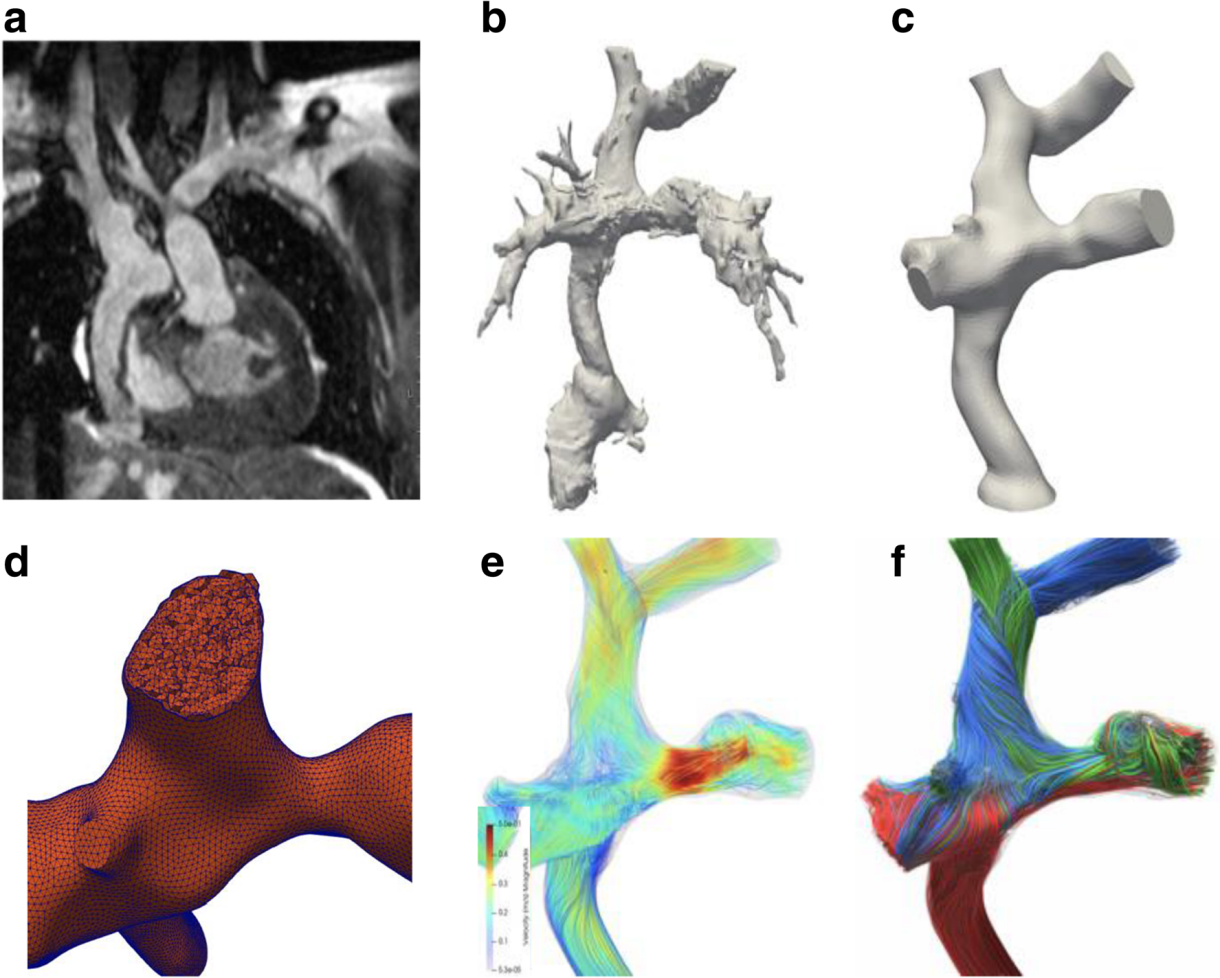
MRI of a Fontan circuit for simulation and prediction of flow. **a–f** MR 3-D steady-state free precession data (**a**) are segmented to create a 3-D volume-rendered/3-D print model (**b** and **c**). Using a reconstruction mesh (**d**) and flow data at specific locations enables the creation of streamlines of flow direction and velocity (red = high velocity and blue = low velocity; **e** and **f**), and the possibility to simulate changes of vessel diameters and create virtual flows. Figures courtesy of Jan Ludvig Vinningsland of the Norwegian Research Centre, Norway

Cardiac MRI is the standard for reliable serial evaluation of ventricular performance, valve function and flow data, with the added possibility of an anatomical overview with multiplanar and volume-rendered reconstructions (Fig. 6). Contrast media based on gadolinium or ferumoxytol can enhance the morphology, enabling 3-D print models of the heart defect [35, 36]. Functional flow data can be provided by 2-D phase-contrast sequences, while newer quantitative MR techniques like 4-D flow with computational fluid dynamics are under research and in clinical use in a few institutions and require specific software analysis tools (Fig. 8) [37–39]. MR strain analysis and feature tracking are, like echocardiography, newer techniques that are garnering more attention to how diastolic and systolic function perform in the single-ventricle circulation and also in pediatric patients [28, 34].

**Fig. 9:**
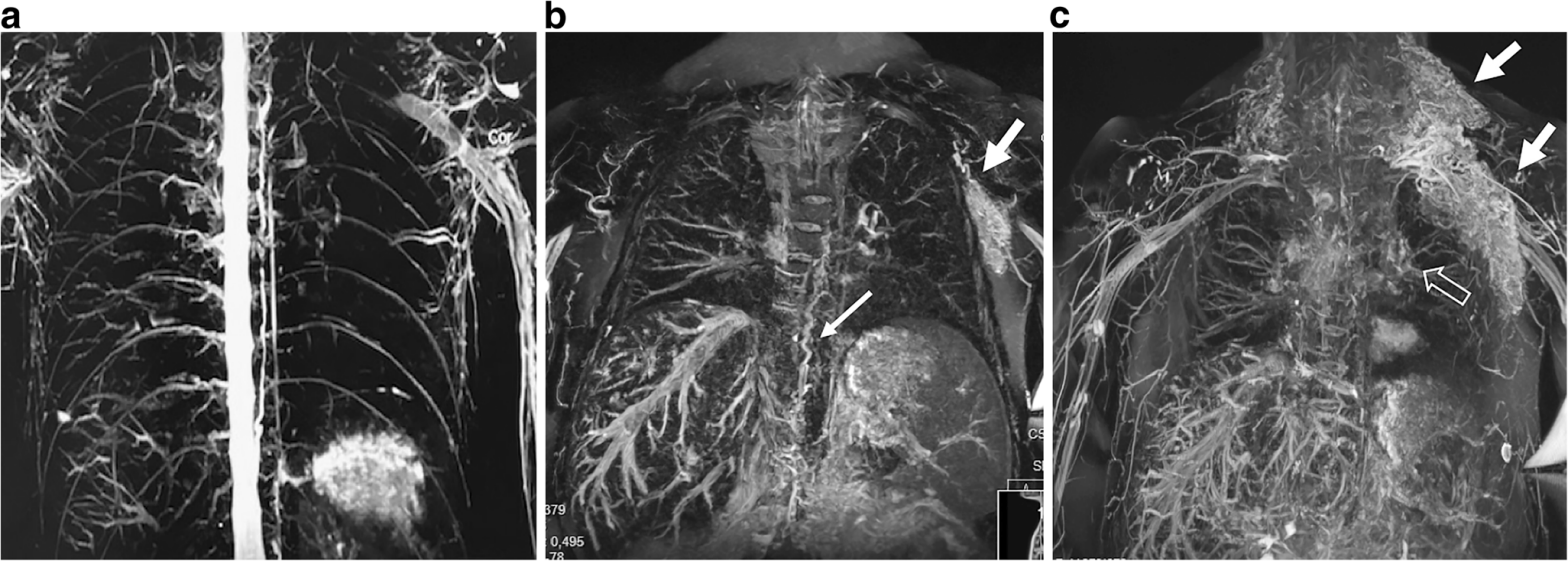
Magnetic resonance lymphography. **a–c** T2-weighted coronal MR sequence in a healthy control subject, a 25-year-old woman (**a**), and in a Fontan-operated 16-year-old boy (**b, c**) with findings of a tortuous thoracic duct (*thick arrows* in **b**). Note the lymphangiectasis in the supraclavicular and axillar regions on the left side (*solid arrows* in **b** and **c**) and perihilar region (*open arrows* in **c**). There is additional perivenous edema in the liver and mesentery (**b**)

Magnetic resonance imaging offers the possibility for tissue characterization by relaxometry with T1, T2 and T2* mapping of the myocardium for evaluating evidence of myocardial edema, scarring, diffuse fibrosis and iron deposition [40, 41]. In addition, visualization of the lymphatic system in the thorax with MR lymphography can be employed with and without intravenous contrast agent (Fig. 9) [42]. Many of the new quantitative techniques in MR have only been used in smaller studies in pediatrics and are still under research to provide evidence-based data to prove their usefulness.

Cardiac CT angiography is especially important for coronary artery visualization and in children with MR-incompatible devices or who are not clinically able to undergo a cardiac MRI (Fig. 6). Especially in pediatrics, scanning protocols with radiation-reducing techniques must be used. For Fontan circulation, specific contrast-injection protocols are necessary depending on the clinical question. Knowledge of pitfalls in the interpretation is important [33, 43] (Fig. 7). Functional cardiac information can also be provided by electrocardiography-gated scanning of the whole cardiac cycle at the cost of increased radiation and performed in select cases, such as children with an implantable device like a cardioverter defibrillator [33, 44]. Both MR and CT are used to guide intervention for catheter-based ablation of arrhythmia [45, 46].

Cardiac catheterization provides, invasively, a direct hemodynamic evaluation and the possibility for interventional procedures to relieve and optimize central venous pressure in pulmonary artery stenosis and to treat venovenous or arteriovenous collaterals (Fig. 6). Evaluation before the final Fontan stage of anatomical and functional features is often performed using echocardiography and catheterization in combination. However, many centers prefer cardiac MRI because it is noninvasive, without ionizing radiation, and it provides additional important morphological information and more reliable volumetric measurements than echocardiography [47–49].

## Fontan-associated liver disease

Fontan-associated liver disease is an inevitable development after Fontan palliation and is a growing concern for these young patients. Although liver injury caused by cardiac dysfunction has long been recognized [50], in Fontan patients, the chronic elevated central venous pressure without pulsations, lymphatic overflow and hypoxia-induced sinusoidal stress results in fibrosis/cirrhosis with special features where the pathophysiological mechanisms are still not fully understood. Fibrosis can be seen in the first 5 years after Fontan completion [6, 51] and develops in different phases during adolescence/adulthood to end-stage chronic liver disease, where most complications occur, such as portal hypertension with variceal bleeding, ascites, hepatic encephalopathy and hepatocellular carcinoma [6, 8, 23]. Typically, hepatic serological markers are normal or only slightly deranged, with increase in transaminases, glutamyl transferase and bilirubin and with no or few clinical findings until the more advanced stages or development of hepatocellular carcinoma, where serum alpha-fetoprotein might be increased [52]. The venous hypertension mimics a hepatic venous outlet obstruction and gives rise to arterialization of hepatic blood flow with presence of hypervascular nodules [53]. Other types of nodules develop with or without signs of cirrhosis, with hepatocellular carcinoma reported in 1.3% of Fontan patients as young as 12 years [23, 54].

The American College of Cardiology statement on Fontan-associated liver disease and several other reports and researchers advocate for periodic radiologic liver assessment screening and longitudinal follow-up of fibrosis. In addition, there is a need for a workup and a surveillance plan for malignant transformation in the presence of larger nodules [6, 52, 55]. Monitoring liver disease development is important for medical and interventional treatment to optimize the Fontan circulation and to identify the optimal time point for cardiac transplantation [56].

**Fig. 10:**
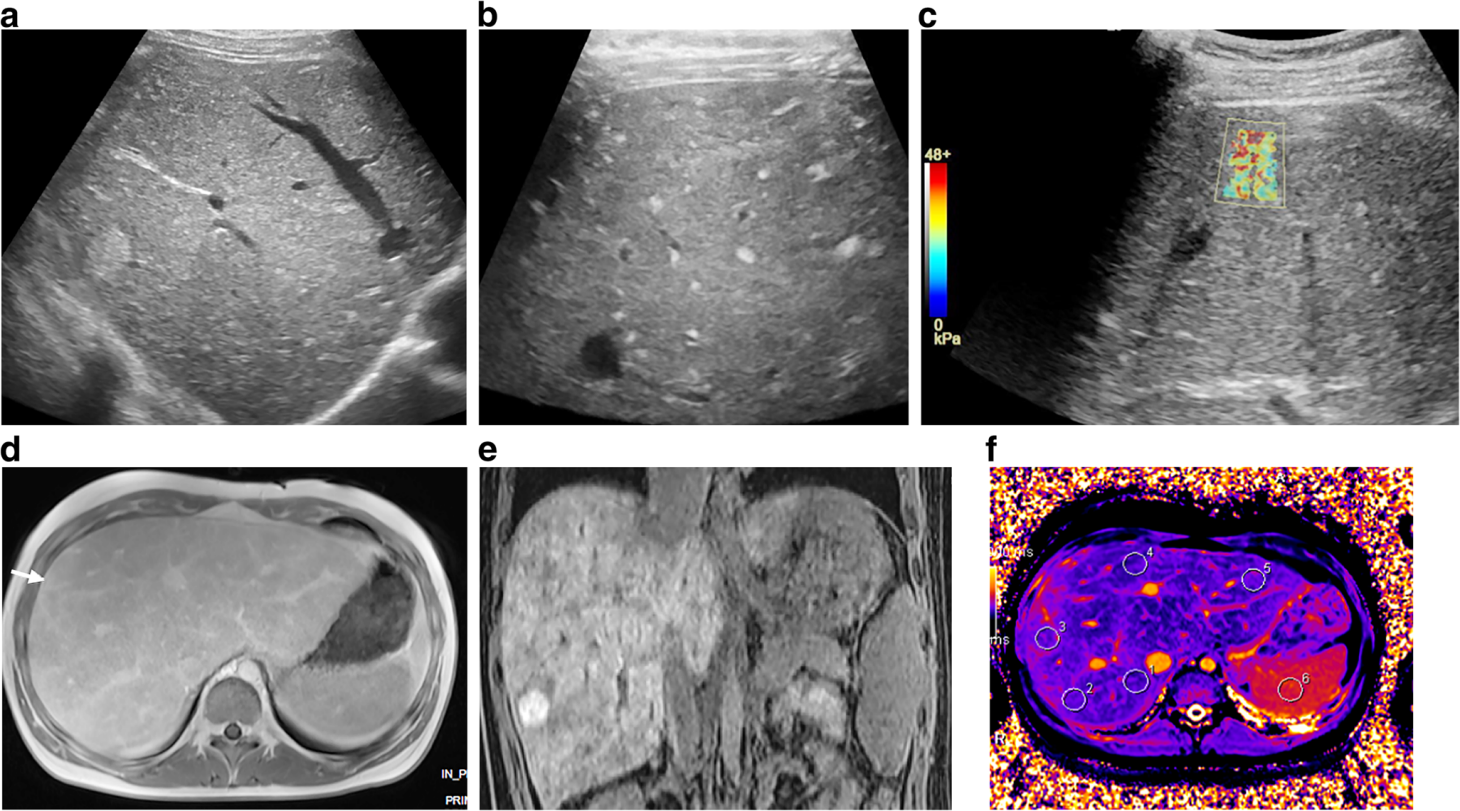
Ultrasound elastography and MRI in Fontan-associated liver disease in a 16-year-old girl with fibrosis, cirrhosis and nodular liver lesions. **a** Sagittal US imaging reveals a heterogeneous parenchyma and a large nodular lesion of 2 cm in diameter laterally in the right liver lobe. **b, c** Transverse linear high-frequency US probe reveals multiple small hyperechoic lesions (**b**), and transverse shear-wave elastography shows increased liver stiffness (**c**). **d** Axial T1-W gradient echo MR image post gadolinium contrast injection reveals an enlarged congestive liver with irregular enhancement of the nodule (*arrow*). **e** Coronal T1 gradient echo image shows strong enhancement of the nodule with gadoxetic acid in late hepatobiliary phase, interpreted as a focal-nodular-hyperplasia-type lesion. **f** MR native T1 mapping in an axial midsection plane, with regions of interest drawn in the liver and spleen, shows irregular increased T1 times in the periphery

Ultrasonography has been proposed as a first choice of investigation, although it is relatively insensitive in detecting fibrotic changes and can miss nodules, which are often isoechoic with the liver parenchyma [57]. US imaging can, however, identify early phases of congestive hepatopathy and provide hemodynamic information on hepatic flow, important for serial evaluation of fibrosis/cirrhosis development. US elastography, especially shear-wave techniques, is gaining more experience in the serial follow-up in these patients [57, 58]. Increased liver stiffness is present in the immediate Fontan postoperative period and persists in the majority of patients (Fig. 10) [59]. Nonetheless, elastography results must be interpreted with caution in the Fontan population because, unlike in other forms of chronic liver disease, no validated cut-offs of severe liver fibrosis are available, especially in pediatrics [55, 60].

Use of contrast-enhanced US imaging in the liver in Fontan patients has not been reported. Although the second-generation US contrast agents like SonoVue (Bracco, Milan, Italy) are approved for pediatric use to diagnose liver lesions in the United States, off-label use in Europe needs careful consideration. Low frequency of adverse effects is reported but the fact that many Fontan patients have serious cardiac dysfunction and venovenous shunting via collaterals could be a contraindication for use [61, 62].

**Fig. 11:**
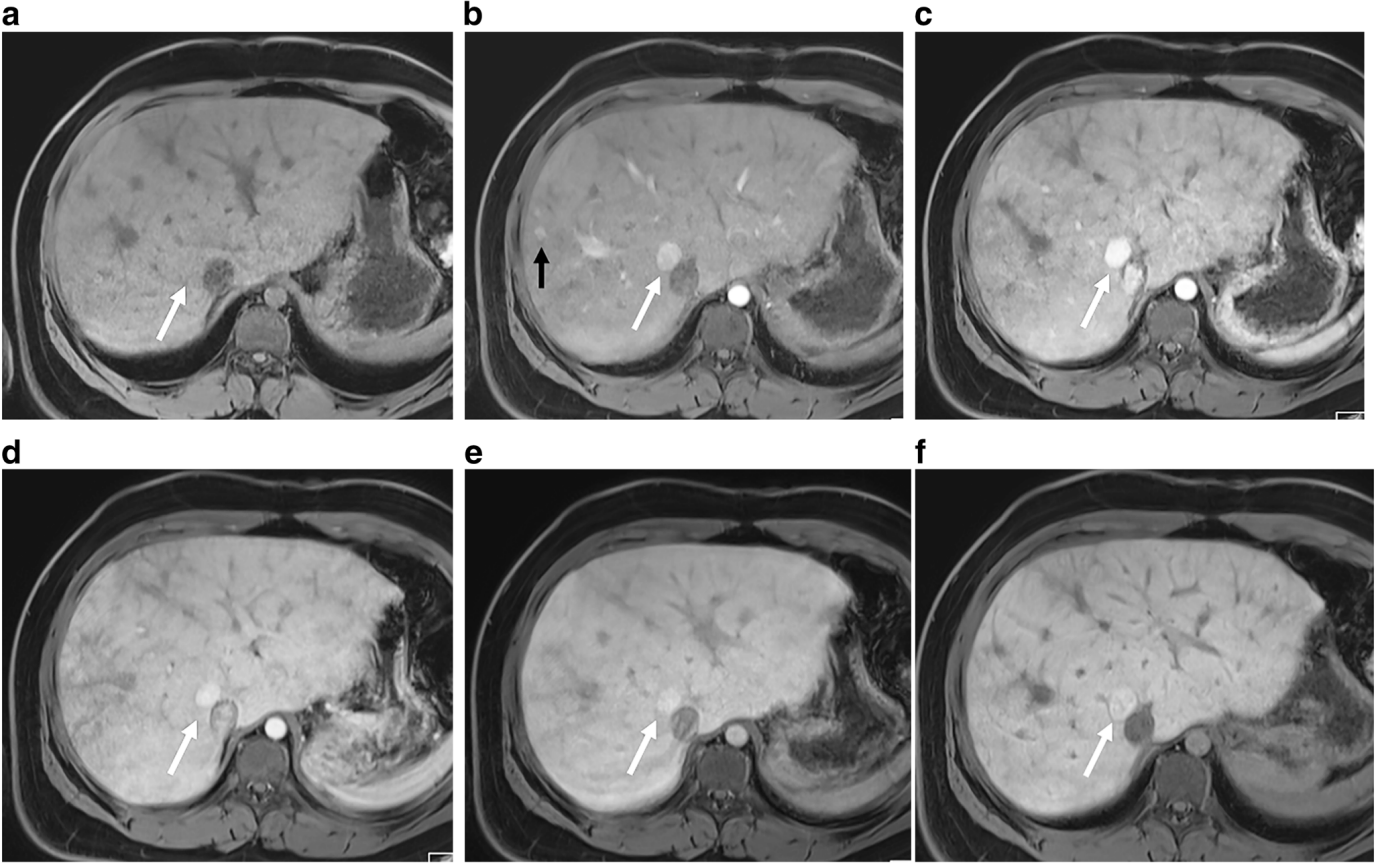
Tricuspid atresia and transposition of the great arteries in a 15-year-old girl with Fontan circulation and normal hepatic serological markers. As part of regular follow-up, MRI was performed including an axial gradient echo breath-hold sequence with fat suppression, sequences pre- (**a**) and post-injection of gadoxetic acid in arterial phase (**b**), portal venous phase (**c**), and after 2 min (**d**), 5 min (**e**) and 27 min (hepatobiliary phase) (**f**). Typical findings show an enlarged liver with an irregular congestive pattern. There is a nodular lesion anterolateral to the inferior caval vein (*white arrows*) with isointense signal to liver on T1-W pre-contrast and arterial enhancement with partial washout (**e**) and in hepatobiliary phase, a clear enhancement (**f**). The lesion was interpreted as a focal-nodular-hyperplasia-like lesion. Note additional small hypervascular nodules in the periphery (*black arrow* in **b**)

Computed tomography and MRI provide more detailed and accurate complementary information on structure and morphology of the liver tissue and characterization of the liver nodules in different vascular phases, and for MRI, hepatocyte-specific contrast agents can also be used (Figs. 6, 10 and 11). The liver congestion makes the interpretation of nodular vascular enhancement difficult. For diagnosis of hepatocellular carcinoma, the imaging criteria proposed in the Liver Imaging Reporting and Data System (LIRADS) are less reliable [55, 63]. Therefore, contrary to recommendations in adults with nodules suspicious for malignancy, biopsy is suggested in Fontan patients to verify the diagnosis [7, 55]. The role of liver biopsy as routine in the staging of fibrosis/cirrhosis is under debate. Although considered the gold standard in diagnosing cirrhosis, it is an expensive, invasive procedure with risks of complications and sampling errors because of the heterogeneous distribution in this type of liver disease [7, 52, 64, 65].

Other techniques including diffusion-weighted imaging, MR elastography, and relaxometry with T1, T2 and T1rho mapping have been reported to be useful in the diagnosis and in assessing the central venous pressure (Fig.10) [66–68]. US/MRI shear-wave elastography cannot distinguish hepatic congestion from fibrosis but the composite of these two measures might be of important clinical value in the longitudinal follow-up of Fontan-associated liver disease [55, 58].

The cardiac Fontan surgery leads to acute kidney injury. In addition, the kidneys are susceptible to decreased cardiac output as well as possible nephrotoxic medication, cardiopulmonary bypass runs (inflammation), intravenous iodinated contrast agents and longstanding cyanosis. This Fontan-related renal dysfunction often goes undetected until adolescence and adulthood. Measured renal function, i.e. glomerular filtration as opposed to estimated glomerular filtration, is abnormal in at least 10% of these children, with increasing prevalence in adolescents and adults [5].

Monitoring renal function using US Doppler resistive index is of growing interest as a marker of heart failure and end organ damage in adult congenital heart disease. A recent pediatric Fontan study revealed that a high index >0.81 reflects the severity of heart failure, hepatic and renal function, glucose intolerance and overall mortality [69].

## Veno-lymphatic complications

Both protein-losing enteropathy and plastic bronchitis represent the clinical manifestation of chronic venous congestion induced by lymphatic insufficiency and abnormal lymphatic channels [70]. Protein-losing enteropathy affects 3–18% of Fontan patients and is a life-threatening disease when proteins leak into the small bowel [22, 60]. The pathophysiological mechanisms of protein-losing enteropathy in Fontan circulation are incompletely understood and seem to differ compared to other forms. In a recent study by Rodriguez et al. [60], protein-losing enteropathy seemed to be associated with advanced liver disease and markers of inflammation and intestinal permeability. Protein-losing enteropathy diagnosis is made by the evidence of elevated fecal α1-antitrypsin in combination with serum hypoalbuminemia and clinical edemas.

Plastic bronchitis is reported to occur in <5% of individuals with Fontan circulation and consists of thick tenacious casts of lymphatic protein-rich fluid from broncho-lymphatic communications, produced within the bronchial tree. Plastic bronchitis is diagnosed after detection of these casts by patient expectoration or by bronchoscopic removal. The casts can partially or completely obstruct the airway, leading to severe hypoxemia and asphyxiation.

The relationship between lymphatic congestion and end organ damage such as liver fibrosis, protein-losing enteropathy and Vitamin D insufficiency and nutrition needs to be further explored. Both plastic bronchitis and protein-losing enteropathy are challenging to treat medically and until recently there was only one option, cardiac transplantation. Nevertheless, recent advances with surgical and interventional procedures have been quite successful, although with a risk of recurrence because these interventions do not treat the Fontan circulation itself and the inherent venous hypertension [71].

Imaging has quite recently gained an important role to investigate and tailor interventional treatment. Using MR lymphography based on heavily T2-weighted sequences, patterns of lymphatic vascular architecture in the chest and abdomen can be delineated (Fig. 9). When performed in conjunction to the Fontan completion, these patterns have been shown to predict the outcome with high association to protein-losing enteropathy and plastic bronchitis [72]. New treatment options, including surgical and catheter-based intervention to occlude or stent vasculo-lymphatic or bronco-lymphatic pathways, have been successful in small series with good outcomes as well as low morbidity and mortality [42, 73]. Lymphatic conventional angiography and intervention might also be an option in tertiary and specific centers, even with intrahepatic MR lymphography reported [71].

## Bone health and growth

Fontan patients have impaired growth with lower stature compared to the normal population.

Decreased Vitamin D levels and increased parathyroid hormone, low lean muscle mass and decreased cortical bone mineral density are key findings in this patient group [5]. In a recent study, bone mineral density levels decreased with age in people with Fontan circulation where different bone components were involved. Vitamin D levels also decreased with age, but this was not consistently associated with bone mineral density [74]. The reason for the decrease of Vitamin D in Fontan patients is unknown but is thought to be related to altered gut circulation and Vitamin D absorption.

Radiographic bone age estimation, especially using automated techniques in addition to bone mineral density measurements like dual-energy X-ray absorptiometry, can be performed to guide treatment. Dual-energy X-ray absorptiometry is of limited value in pediatrics because of the lack of pediatric normal reference values of bone mineral density in the younger age group [75].

## Central nervous system and neurocognitive function

There is increasing awareness of neurologic, neurodevelopmental and behavioral abnormalities in congenital heart disease, and especially in Fontan patients. The potential causes of brain injury in congenital heart disease are cumulative and interactive. There are genetic causes, with in utero disturbed hemodynamics that can reduce oxygen delivery and cerebral maturation. Other factors are patient-related, like low birth weight and preterm birth. The different steps of surgical treatment and cardiac catheterization contribute to the risk of hypoxic–ischemic injury and embolic events [25]. Neurocognitive impairment impedes academic achievement and evaluation in early school years. There is a need to target patients who might benefit from interventions. Improving the understanding of how to manage cognitive and psychiatric outcomes in adult congenital heart disease, especially Fontan patients, is a prioritized area of research.

Fetal and neonatal MR studies in children with a single ventricle have shown abnormalities in maturation, volumetric growth and white matter already in fetal life [76–79]. MR findings in later life show striking deviations from normal in most people, with focal and multifocal abnormalities of different degrees. Findings show that non-acute ischemic changes such as atrophy and ventriculomegaly accumulate with staged surgical reconstruction [80]. Typically, spreading white matter abnormalities have been associated with cognitive impairment as well as gray matter abnormalities [81, 82].

## Heart transplantation

The only curable treatment for children with Fontan circulation is a heart transplantation. There is an ongoing debate on the optimal time point to perform a transplantation. Many children reach such a serious reduced clinical state before considering transplantation that the procedure itself becomes too dangerous regarding pre-, peri- and postoperative complications [83]. Ventricular assist devices might be necessary to bridge a cardiac transplantation in trying to achieve a better clinical and functional state [84]. Advanced liver cirrhosis and even malignant transformation might prohibit transplantation or require a combined liver and heart transplantation [6]. There has been a high early post-transplantation mortality rate in this patient group, but with better perioperative treatment, survival is increasing and is now comparable to the rate in other types of congenital heart disease undergoing transplantation [85].

Pre- and post-transplantation imaging consists of chest radiography, CT and cardiac MRI to delineate morphological and functional features that are important for the choice of surgical approach and post-transplant complications [86].

## Radiologic imaging in the surveillance of Fontan patients

What is the role of imaging in the surveillance of this patient group? No or very few evidence-based studies exist because of the still quite short observational period, and the proposed published guidelines until now were mainly based on experience in the adult age group. When a pediatric cardiologist follows a Fontan patient, his or her personal knowledge of how the other organs are affected will guide the surveillance by other clinical specialties. When these children reach early adolescence, their transition and follow-up from pediatric to adult care regarding both cardiology and radiology needs to be assured until successful commitment [9].

A statement from the American Heart Association from 2019 acknowledged that there is insufficient evidence base for developing clear recommendations but that cardiac and end-organ surveillance in children and adults is warranted and clinically important [5]. Furthermore, the statement said that monitoring should be performed by multidisciplinary Fontan/single-ventricle care clinics with health care personnel familiar with and experienced with Fontan circulation and complications [5]. It is expected that a surveillance protocol — the frequency and type of tests, including imaging — will, with the increased knowledge and experience from multicenter studies, be transformed in the future. Cardiac and end-organ imaging has an important part as a supplement to clinical assessment and other tests and should be carried out at defined intervals as proposed [5] and as discussed in this section.

Cardiovascular imaging assessment is already common at many institutions and should be performed in all age groups at set intervals. Cardiac MR with anatomical and functional investigation should be performed every 2–3 years and CT angiography when clinically indicated [47]. Cardiac catheterization is recommended once every 10 years or when clinically indicated [28].

End-organ status assessment is a newer concept where imaging and frequency should be tailored to patient age — every 3–4 years for children ages <12 years and every 1–3 years for adolescents ages 12–18 years. Findings at any test might change the interval for follow-up or initiate other investigations. The general imaging strategy should be as follows:

- Lymphatic and endocrine system: MR lymphography with T2-weighted MR imaging as a new technique is recommended as likely to be of value as a part of a cardiac MR examination. Bone-age estimation and bone-mineral-density assessment are also recommended.
- Brain and neurocognitive function: In addition to neurodevelopmental/cognitive testing, a brain MR examination can provide important information.
- Lungs: Chest radiography is recommended.
- Liver*:* Abdominal/liver US is recommended, including elastography if possible. MRI including liver biopsy might be considered. In the adolescent, this becomes even more important, especially monitoring liver disease development, because advanced-stage cirrhosis or even hepatocellular carcinoma might preclude transplantation. Annual US imaging with elastography has been proposed for adolescents ages 15–16 years, with more frequent controls if radiologic signs of fibrosis and nodules are found [52]. Some centers advocate annual liver MR from adolescence on, later combined with US elastography when routine biopsy is proposed [6, 7, 52].

However, the optimal management strategy for the pediatric population remains to be established [54].

## Conclusion

Although the Fontan procedure has extended patients’ lifespan, an important risk of morbidity and mortality remains. Children receiving surgical treatment with a Fontan procedure at the time of birth require lifelong follow-up with medical care, implicating an important financial and societal impact. Still, much is unknown of how the Fontan palliation affects patients differently. End-organ effects are increasingly recognized, where mainly the cardiac, lymphatic and liver changes require monitoring with radiologic techniques to ensure the optimal timing for treatment and eventually cardiac transplantation. Increasing knowledge in pediatric radiology is urgently needed regarding this growing patient group and the pathology in end organs in addition to the cardiac changes.

## Electronic supplementary material

ESM 1 (MP4 3,661 kb)
